# Paeoniflorin exerts a nephroprotective effect on concanavalin A-induced damage through inhibition of macrophage infiltration

**DOI:** 10.1186/s13000-015-0347-4

**Published:** 2015-07-25

**Authors:** Cheng Liu, Zhuoan Cheng, Yunman Wang, Xiuqin Dai, Jie Zhang, Dongying Xue

**Affiliations:** Experimental Research Center, Putuo Hospital, Shanghai University of Traditional Chinese Medicine, Shanghai, 200062 China; Department of Nephrology, Putuo Hospital, Shanghai University of Traditional Chinese Medicine, Shanghai, 200062 China; Department of Infectious Disease, Putuo Hospital, Shanghai University of Traditional Chinese Medicine, Shanghai, 200062 China

**Keywords:** Paeoniflorin, CXCR3-CXCL11, Macrophage infiltration, Concanavalin A

## Abstract

**Background:**

It is well established that macrophage infiltration is involved in concanavalin A (conA)-induced liver injury. However, the role of macrophages in conA-induced renal injury remains unknown. The aims of this study were to investigate macrophage infiltration in conA-induced renal injury and determine whether paeoniflorin (PF) could inhibit macrophage infiltration into the kidney.

**Methods:**

BALB/C mice were pre-treated with or without PF 2 h (h) before conA injection. At 8 h after con A injection, all the mice were sacrificed; The liver and kidney histology were studied. The renal CD68 expression was detected by immunohistochemical and real-time PCR analysis. The level of expression of C-X-C chemokine receptor type 3 (CXCR3) was analyzed by western blot, immunohistochemical and real-time PCR. The pathophysiological involvement of CXCR3 in macrophage infiltration were investigated using dual-colour immunofluorescence microscopy.

**Results:**

PF administration significantly reduced the elevated serum levels of alanine transaminase (ALT), blood urea nitrogen (BUN), creatinine (Cr) and the severity of liver and renal damage compared with that in the conA-vehicle group. PF administration inhibited the increase in renal IL1β mRNA expression and concentration. Furthermore, immunohistochemical analysis showed that macrophages secreted CXCR3 in the kidneys of the conA-vehicle mice. Immunofluorescence microscopy demonstrated CXCR3 bound tightly to C-X-C motif ligand 11 (CXCL11) in the kidneys of the conA-vehicle mice and showed that PF treatment could suppress CXCR3/CXCL11 over-activation.

**Conclusions:**

Macrophage infiltration was a notable pathological change in the kidneys of conA-treated mice. PF administration attenuated conA-induced renal damage, at least in part, by inhibiting the over-activated CXCR3/CXCL11 signal axis.

## Background

ConA-induced liver injury is a well-established mouse model used to detect immune cell-mediated acute hepatitis, and closely resembles the pathology of human autoimmune hepatitis [1]. ConA-induced liver injury is characterised by a marked increase in plasma alanine transaminase (ALT) levels from 8 to 24 h after injection, as well as simultaneous hepatic infiltration by CD4^+^ T immune cells, natural killer T cells (NKT), Kupffer cells, and neutrophils [2]. The activation of lymphocytes and production of inflammatory cytokines and chemokines are well studied in conA-induced liver injury [3, 4]; however, it remains unclear whether the kidney is damaged in conA-treated mice.

Paeoniflorin (PF), a monoterpene glycoside, is the principal bioactive component of *Radix Paeoniae Rubra*, a traditional Chinese herbal medicine [5]. PF inhibits hepatocyte apoptosis and the potential mechanism underlying this is associated with the regulating mediators in endoplasmic reticulum (ER) stress and mitochondria-dependent pathways [6]. PF reduces dextran sodium sulphate (DSS)-induced colitis by suppressing the expression of toll-like receptor 4 (TLR4) and reducing the activation of nuclear factor-kappa B (NF-κB) and mitogen-activated protein kinase (MAPK) pathways [7]. PF has also been used for the treatment of liver fibrosis induced by the *Schistosomiasis japonica* egg [8]. We have recently reported that PF inhibits liver fibrosis induced by dimethylnitrosamine (DMN) in rats [9]. Renal macrophages, similar to hepatic Kupffer cells, increased significantly after two weeks of DMN treatment, then decreased after four weeks of DMN administration. Therefore, PF could inhibit renal macrophage activation in DMN-induced liver fibrosis. As a result, it has been hypothesised that the kidney is damaged in conA-induced hepatitis, and PF could reduce conA-induced renal damage by inhibiting macrophage infiltration. It was investigated 1) whether the kidney was damaged, and if so, the macrophage involvement was assessed; 2) whether PF reduced renal damage and macrophage infiltration in conA-induced injury; and 3) whether the CXCR3/CXCL11 signalling pathway was involved in macrophage infiltration in conA-induced injury. This study describes a newly discovered effect of PF and a previously unknown functional mechanism in renal diseases.

## Methods

### Major materials

Paeoniflorin (PF, >95 % purity), DAPI fluorescent stain, and conA type IV were obtained from Sigma (St Louis, MO, USA). The SABC kit for immunohistochemical analysis was obtained from Boster (Wuhan, China). The IL1β ELISA kit was from R&D system (Minneapolis, MN, USA). The antibodies used for the immunohistochemical and western blot analyses were rabbit polyclonal IL1β (sc-7884), goat polyclonal monocyte chemotactic protein 1 (MCP1) (sc-1785), rabbit polyclonal F4/80 (sc-25830), mouse monoclonal CXCR3 (sc-137140) and rabbit polyclonal CXCL11 (sc-28874) purchased from Santa Cruz Biotechnology (La Jolla, CA, USA). Mouse monoclonal CD68 (MCA31R) was obtained from Serotec (Oxfordshire, OX51GE, UK). Secondary fluorescence-labelling goat anti-mouse Cy3 and goat anti-rabbit FITC second antibodies were obtained from Jackson (West Grove, PA, USA).

### Ethics statement

All of the study protocols complied with the current ethical considerations of Shanghai University of Traditional Chinese Medicine’s Animal Ethic Committee and the procedural and ethical guidelines of the Chinese Animal Protection Act, which is in accordance with the National Research Council criteria. All animal experiments and procedures were reviewed and approved by the Institutional Animal Care and Use Committee (IACUC) of Shanghai University of Traditional Chinese Medicine and were performed in accordance with the relevant guidelines and regulations.

### Animals

60 Female BALB/C mice at (18 ± 2 g) were supplied by the Central Animal Care Facility of Shanghai University of Traditional Chinese Medicine and housed in an air-conditioned room at 25 °C with a 12 h darkness/light cycle. The mice received humane care with unlimited access to food and water during the study.

### ConA-induced tissue damage in mice

Mice received conA injection via the tail vein at 15 mg/kg body weight. PF (6 mg/kg, 30 mg/kg, or 150 mg/g) was orally administered 2 h before conA injection and control mice received vehicle (distilled water) or PF (30 mg/kg). There was 10 mice in each group. At 8 h after conA injection, all the mice were euthanized under 2 % pentobarbital sodium, and all efforts were made to minimise suffering; kidney and liver samples were taken for the following investigations.

### Histology analysis

The kidney and liver specimens were preserved in 4 % paraformaldehyde and dehydrated in a graded alcohol series. The specimens were embedded in paraffin blocks, cut into 3 μm-thick sections, and placed on glass slides. The sections were then stained with haematoxylin-eosin (HE).

### Liver and kidney function tests

Serum levels of ALT, BUN, and Cr were measured in samples obtained at the end of the experiment. Activity and content were evaluated using a commercial clinical test kit (Jiancheng Institute of Biotechnology, Nanjing, China) according to manufacturer’s instructions.

### Measurement of cytokine levels in the kidneys

The kidneys were homogenised in 5 ml ice-cold physiological saline and the supernatant was obtained by centrifugation at 3000 g for 10 min. Samples were analysed and absolute values were obtained by comparison with standards.

### Immunohistochemistry

Embedded tissue was deparaffinised in xylene and rehydrated. Microwave antigen retrieval was carried out for 5 min before quenching the peroxidase with 3 % H_2_O_2_ in phosphate-buffered saline (PBS) for 10 min at room temperature. The sections were blocked using 5 % BSA for 30 min at 37 °C, and then incubated with the respective primary antibodies (anti-CD68, anti-IL1β, anti-MCP1, and anti-CXCR3) at room temperature for 1 h. After washing with PBS, sections were subsequently incubated with species-specific biotinylated secondary antibodies at room temperature for 30 min. After washing, the sections were stained with 3,3′-diaminobenzidine (DAB). Counterstaining was performed with haematoxylin before dehydration and mounting.

### Immunofluorescent staining

Double staining for F4/80 and CXCR3, and, CXCR3 and CXCL11 were performed on paraffin sections. Sections were deparaffinised in xylene, rehydrated and incubated with protease K (20 μg/ml) for 10 min at 37 °C. Thereafter, the slides were incubated with 5 % BSA for 30 min followed by incubation with anti-F4/80 primary antibody at 37 °C for 1 h. Slides were then washed three times with PBS and incubated with the secondary FITC-conjugated Affinipure goat anti-rabbit antibody for 30 min. After washing, the sections were incubated with the CXCR3 antibody followed by Cy3-conjugated Affinipure goat anti-mouse antibody. Nuclei were labelled with DAPI. Imaging analyses were performed using an Olympus (Osaka, Japan) BX43 system.

### Real-time PCR analysis

Total RNA was extracted from kidney tissue using Trizol reagent (Invitrogen, Carlsbad, CA). A high-capacity cDNA reverse transcription kit (Applied Biosystems, Forster City, CA) was used to synthesise the cDNA. PCR amplification was conducted in 10 μl of solution containing 3 μl cDNA, 5 μl SYBR mixture, 1.6 μl H_2_O, and 0.4 μl primer (10 μM). The primers used were as follows: CXCR3, 5′-TCTCGTTTTCCCCATAATCG-3′ (forward) and 5′-AGCCAAGCCATGTACCTTGA-3′ (reverse); CXCL11, 5′-CATTTTGACGGCTTTCATCC-3′ (forward) and 5′-AAGGTCACAGCCATAGCCCT-3′ (reverse). CD68, 5′-ACCGCCATGTAGTCCAGGTA-3′ (forward) and 5′-ATCCCCACCTGTCTCTCTCA-3′ (reverse). MCP1 5′-ATTGGGATCATCTTGCTGGT-3′ (forward) and 5′-CCTGCTGTTCACAGTTGCC-3′(reverse). IL1β 5′ -GGCTCATCTGGGATCCTCTC-3′ (forward) and 5′-TCATCTTTTGGGGTCCGTCA-3′ (reverse).18 S rRNA, 5′-AGTCCCTGCCCTTTGTACAC-3′ (forward) and 5′ -CGATCCGAGGGCCTCACTA-3′ (reverse). Amplification steps consisted of 40 cycles of denaturation at 94 °C for 40 s, annealing at 55 °C for 40 s, and extension at 72 °C for 40 s using a DNA cycler CFX96 real-time system (Bio Rad, Hercules, CA, USA).

### Western blot analysis

Kidney samples were prepared in ice-cold radio-immune precipitation assay (RIPA) buffer with protease inhibitors. Samples were centrifuged for 10 min at 12,000 rpm. The supernatant was collected, and the protein concentration was measured using a BCA commercial kit. Protein lysates were separated by SDS-PAGE and subsequently transferred onto nitrocellulose membranes. Membranes were blocked with 5 % non-fat dry milk buffer and incubated with antibodies against CXCR3 and CXCL11. A secondary antibody was used for chemiluminescent detection. The loading accuracy was evaluated by monoclonal antibody against GAPDH.

### Statistical analysis

Each experiment was performed at least three independent times. All the results are expressed as mean ± s.d. The statistical test was performed with SPSS software version 18.0. Groups were compared using one-way analysis of variance with Dunnett’s multiple comparison test or the Student-Newman-Keuls test. *P* < 0.05 was considered statistically significant.

## Results

### Suppressed conA-induced liver injury in PF-treated mice

**Fig. 1:**
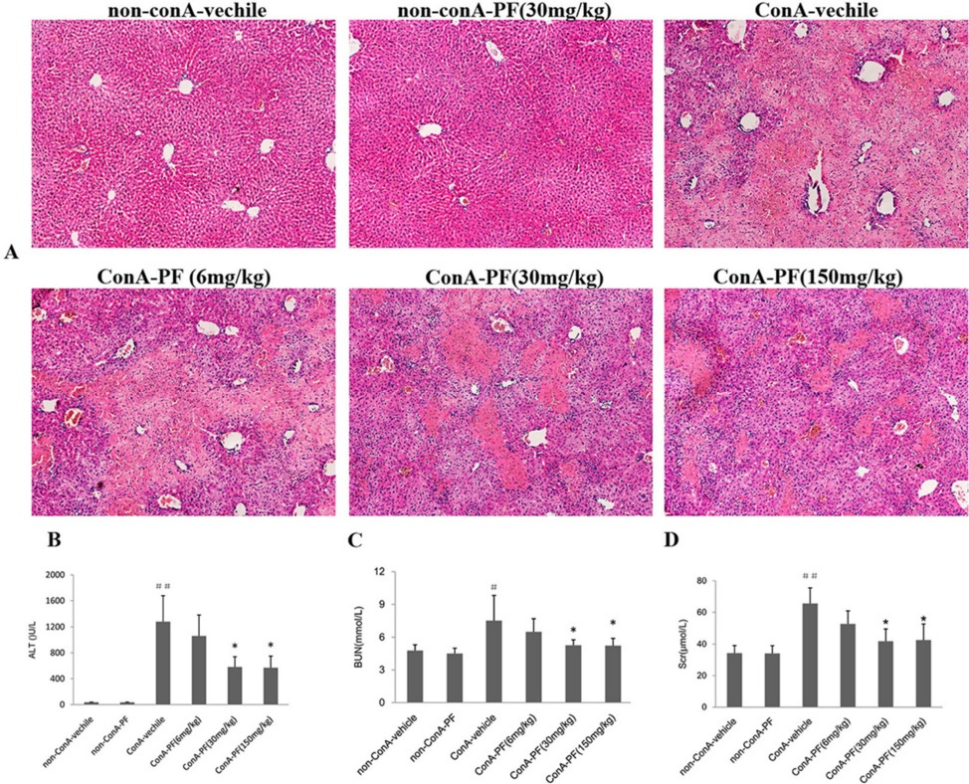
The hepatorenal protective effect of paeoniflorin (PF) in conA-induced mouse model. **a** Effect of PF on light micrographic changes in the liver after conA injection (×100). **b** Effect of PF on serum ALT activity in conA-induced liver injury in mice. **c** Effect of PF on serum BUN and Cr content. **d** In conA-induced liver injury in mice, PF (6 mg/kg, 30 mg/kg, and 150 mg/kg) was administered orally 2 h before injection of conA or vehicle for 8 h. The number in HE staining, serum ALT, and renal function was as the same as the animal number in each group. The data represented the mean ± SD ^#^ *P* < 0.05, ^##^ *P* < 0.01, vs non-conA-vehicle mice, *P < 0.05, * *P < 0.01, *vs* conA-vehicle mice

To test the effects of PF on conA-induced mouse hepatitis, PF (6 mg/kg, 30 mg/kg, or 150 mg/kg) was intraperitoneally administered. Light microscopy examination showed that 8 h after intravenous injection of conA (15 mg/kg body weight) severe liver histopathology was induced, i.e. extensive areas of hepatocyte necrosis and marked sinusoidal hyperaemia associated with haemorrhage. On the other hand, mice pre-treated with PF (30 mg/kg and 150 mg/kg) showed only minor liver damage (Fig. 1a). Serum ALT levels were also measured after conA injection. As shown in Fig. 1b, serum ALT levels were significantly lower in the conA-PF (30 mg/kg and 150 mg/kg) group compared with conA-vehicle mice.

### PF attenuated conA-induced renal damage

To assess kidney damage, renal function and renal histopathological analysis was performed. The levels of serum BUN and Cr increased significantly 8 h after conA treatment (Fig. 1c and 1d). PF (30 mg/kg and 150 mg/kg) showed attenuated renal injury, i.e. decreased serum BUN and Cr levels (Fig. 1c and 1d).

**Fig. 2:**
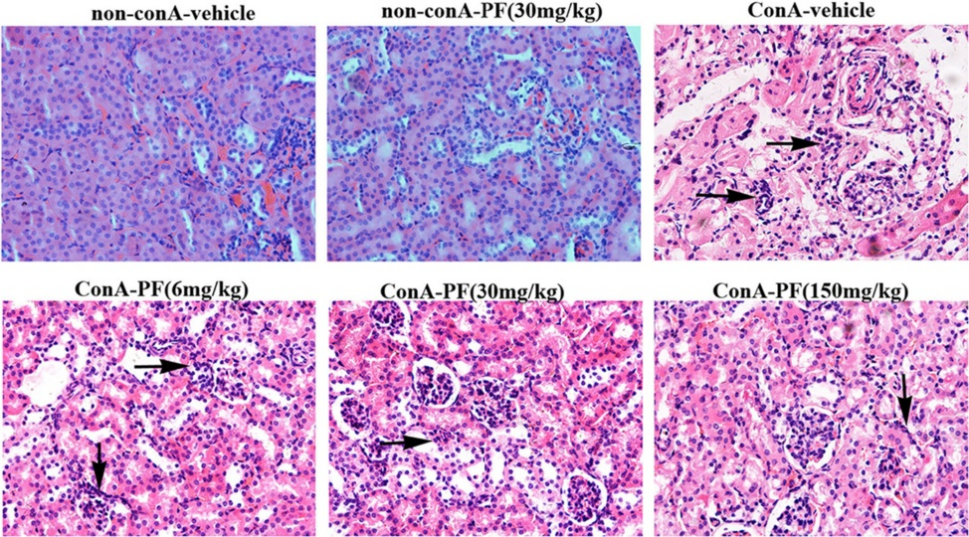
Morphology of kidneys and the protective effect of PF in conA-induced mouse model. Renal HE staining (×400). Arrow means renal injury. PF (6 mg/kg, 30 mg/kg, and 150 mg/kg) was administered orally 2 h before injection of conA or vehicle for 8 h. The number in renal HE staining was as the same as the animal number in each group

Kidney in the non-conA-vehicle and non-conA-PF group showed normal architecture. After 8 h of conA treatment, massive renal tubular epithelial cell necrosis and intense pro-inflammatory cytokine infiltration were found (Fig. 1a). PF (30 mg/kg and 150 mg/kg) showed attenuated tubular epithelial cell necrosis and pro-inflammatory cell infiltration (Fig. 2).

These results suggest that the kidney is damaged after conA administration. PF (30 mg/kg dose and 150 mg/kg) has shown efficacy in conA-induced liver and renal injury in mice. Therefore, PF was selected at a dose of 30 mg/kg for exploring the mechanism of action in the following study.

### PF inhibits renal macrophage and pro-inflammatory cytokines

**Fig. 3:**
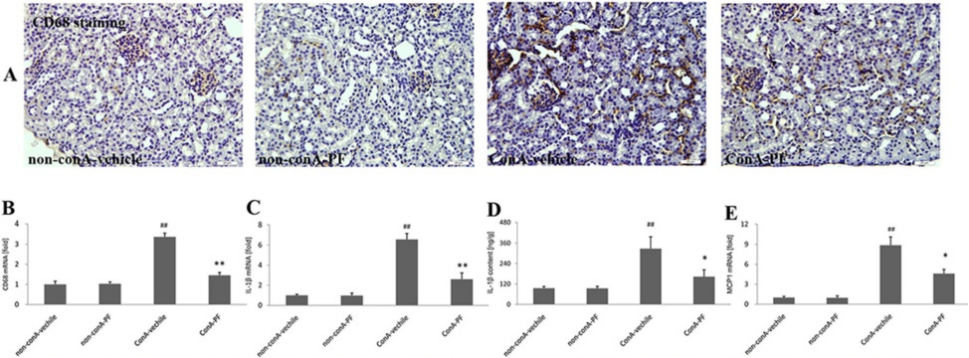
Effect of PF on macrophage infiltration and pro-inflammatory cytokines in conA-induced renal damage. **a** CD68 immunohistochemical staining (×400, n = 3), Brown staining indicates immunopositivity. **b** real-time PCR showing the change in CD68 (n = 6), (**c**) IL1β mRNA expression by real-time PCR analysis (n = 6), (**d**) IL1β content analysis by ELISA analysis, The number in ELISA was as the same as the animal number in each group. **e** MCP1 mRNA analysis by real-time PCR (n = 6). PF (30 mg/kg) was administered orally 2 h before injection of conA or vehicle for 8 h

Macrophage involvement in conA-induced renal injury was assessed. As shown in Fig. 3a, CD68-positive macrophages were at low levels in non-conA kidneys. CD68-positive macrophages, with strong staining, appeared in the renal interstitium and glomerulus in conA-vehicle mouse kidneys. PF pre-treatment could inhibit conA-induced CD68 expression.

Consistent with the results of immunostaining of CD68 (Fig. 3a), increased expression of CD68 was confirmed by real-time PCR analysis (Fig. 3b). Real-time PCR showed that CD68 mRNA was up-regulated around 3.3-fold 8 h after conA treatment. In the conA-PF group, there was a significant reduction in the CD68 mRNA expression compared to the conA-vehicle group (*P* < 0.01).

As presented in Fig. 3c, in conA-vehicle kidneys, the expression of IL1β increased significantly (*P* < 0.01) by more than 6.5-fold compared with the non-conA group. However, PF attenuated IL1β expression compared with conA-vehicle kidneys (*P* < 0.01). This was also confirmed by ELISA (Fig. 3d). In conA-vehicle kidneys, MCP1 mRNA expression increased significantly compared with the non-conA group. PF could inhibit the elevated MCP1 mRNA levels. These results suggest macrophage infiltration into kidneys in conA-induced injury.

### IL1β and MCP1 were derived mainly from renal epithelial cells and not macrophages in conA-induced renal injury

It has been well reported that pro-inflammatory cytokines are derived from macrophages [10], and results in this study showed IL1β and MCP1 increased significantly after conA administration. It was confirmed whether these pro-inflammatory cytokines were produced from the macrophages using immnohistochemical analysis.

**Fig. 4:**
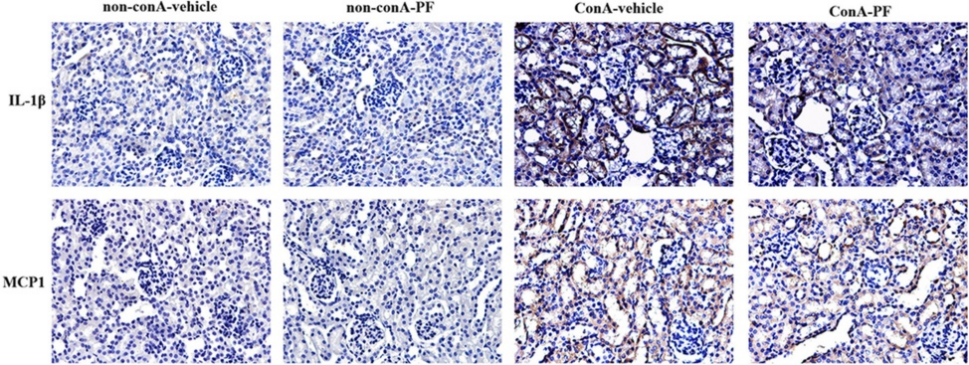
IL1β (*upper*) and MCP1 (*lower*) mostly expressed in tubular epithelial cells in conA-induced renal injury. PF (30 mg/kg) was administered orally 2 h before injection of conA or vehicle for 8 h. (×400, n = 3)

As shown in Fig. 4, tubular epithelial cells were weakly positive for IL1β and MCP1 in non-conA mice. IL1β and MCP1 were located in tubular epithelial cells with strong staining in conA-vehicle mouse kidneys. The strong stained IL1β and MCP1 were suppressed in conA-PF mice. The pro-inflammatory chemokines such as IL1β and MCP1 were almost expressed in tubular epithelial cells, but not in macrophages.

### CXCR3 increased in kidneys after conA administration

**Fig. 5:**
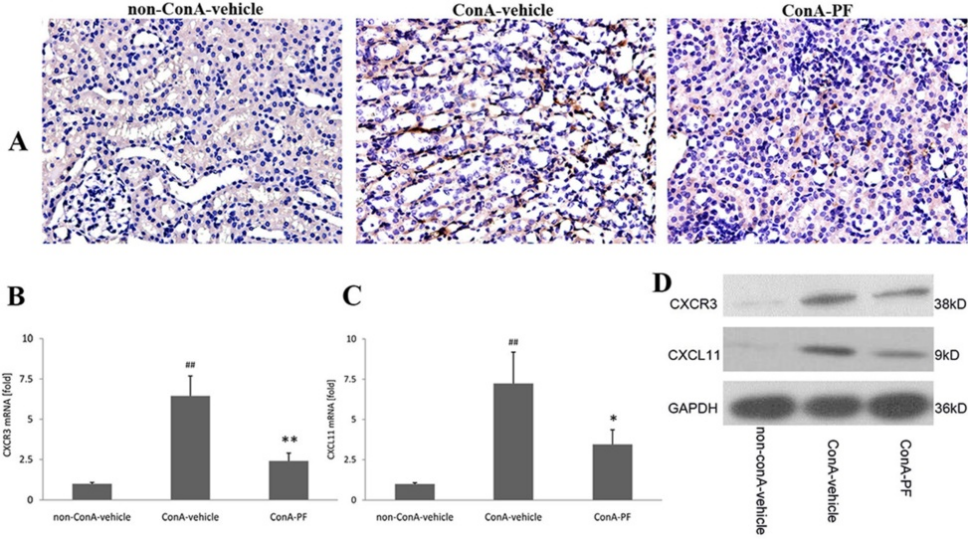
Effect of PF on CXCR3 and CXCL11 expression in conA-induced renal injury. **a** CXCR3 immunohistochemical staining (×400, n = 3). **b** CXCR3 mRNA expression was detected using real-time PCR (n = 6), **c** CXCL11 mRNA expression was detected using real-time PCR (n = 6), **d** CXCR3 and CXCL11 were detected by western-blot (n = 4). PF (30 mg/kg) was administered orally 2 h before injection of conA or vehicle for 8 h

Chemokines play an important role in macrophage accumulation at the inflammatory site. CXCR3 and its ligands have been suggested to be one of the most important chemokine axes that promote the arrival of cells into the injured tissues [11]. As shown in Fig. 5a, CXCR3 was weakly stained in non-conA mouse kidneys. In conA-vehicle mouse kidneys, CXCR3 increased significantly and was largely expressed in the renal interstitium, and not in the tubular epithelial cells. In conA-PF mouse kidneys, CXCR3 was suppressed compared with the conA-vehicle group; this was also confirmed by real-time PCR (Fig. 5b).

The mRNA levels of CXCL11 in the kidneys were assessed using quantitative PCR (Fig. 5c). Higher levels of CXCL11 were recorded in the samples from conA-treated mice than in non-conA samples. In addition, mice in the conA-PF group showed lower CXCR3 levels compared with the conA-vehicle group. This was also confirmed by western blot analysis (Fig. 5d).

### CXCR3 and macrophages co-localise in conA-treated mouse kidneys

**Fig. 6:**
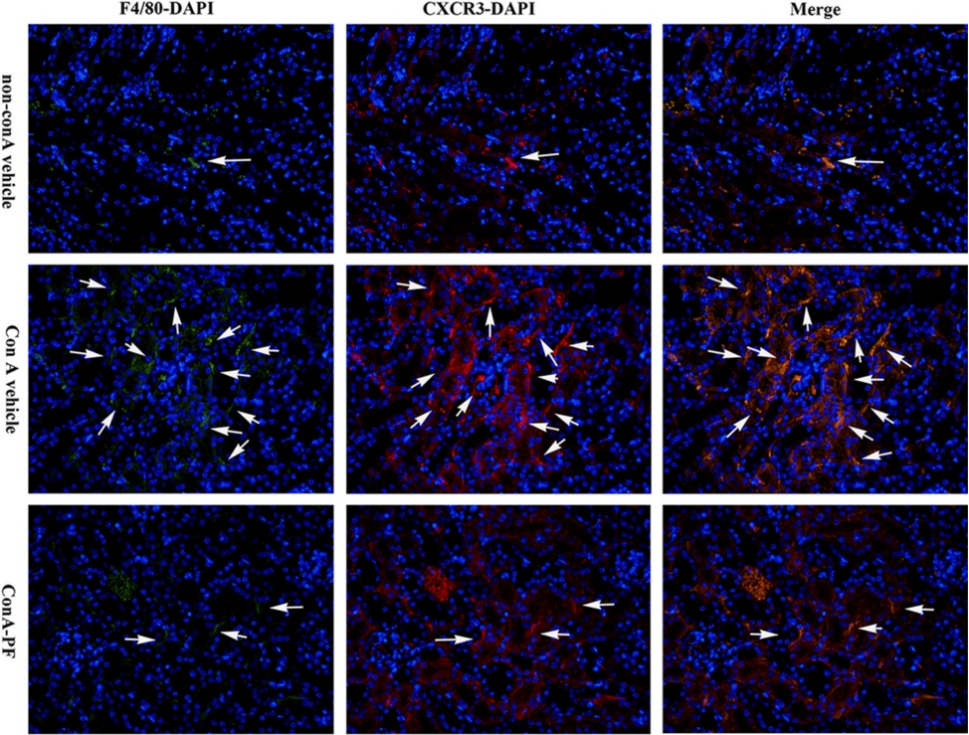
Renal sections were stained using immunofluorescence staining consisting of F4/80 (green), CXCR3 (*red*), and DAPI (*blue*) to determine the relationship between macrophages and CXCR3 expression in conA-induced renal injury. Arrow indicates CXCR3 derived from macrophages (×400, n = 3). PF (30 mg/kg) was administered orally 2 h before injection of conA or vehicle for 8 h

CXCR3 originated mostly from interstitial cells. CXCR3 can also originate from macrophages and plays an important role in conA-induced renal damage. To this end, immunofluorescence analysis was performed. Double staining for F4/80 and CXCR3 showed that F4/80-positve macrophages were producers of CXCR3 in conA-treated kidneys. As expected, there were a few numbers of double F4/80 and CXCR3-positive cells (Fig. 6). Almost all F4/80-positive macrophages were found to be CXCL11-positive. In contrast, a lower number of infiltrating F4/80-positive macrophages, that also had lower expression levels for CXCR3, was observed in the kidneys of conA-PF mice. These results showed that macrophages are the main producer of CXCR3 in conA-induced renal injury.

### Interactions of CXCR3 and CXCL11 observed in conA-treated mouse kidneys

**Fig. 7:**
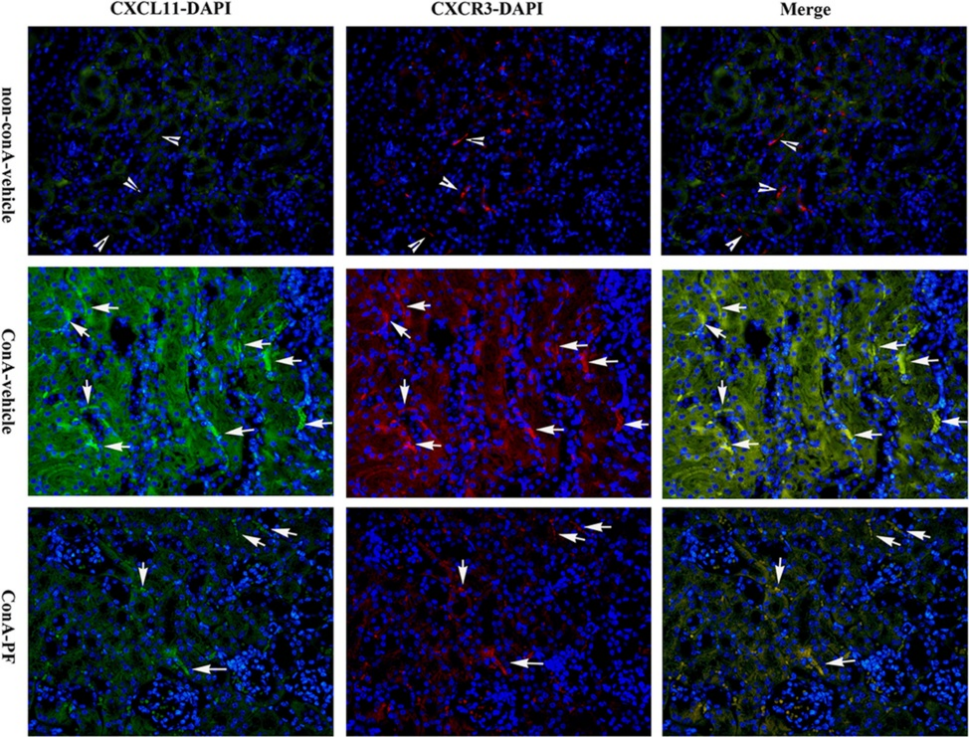
Renal sections were stained CXCR3 (*red*), CXCL11 (*green*), and DAPI (*blue*) in non-conA-vehicle, conA-vehicle and conA-PF mice. Arrowhead indicates there is no interaction between CXCR3 and CXCL11. Arrow indicates binding of CXCR3 and CXCL11 and interactions (×400, n = 3). PF (30 mg/kg) was administered orally 2 h before injection of conA or vehicle for 8 h

Because the number of macrophages is characteristically increased in the conA-vehicle kidneys and macrophages express CXCR3, it was hypothesised that the overproduction of CXCL11 may be involved in macrophage recruitment to the injured kidneys in conA-treated mice. To this end, the presence of CXCR3 with CXCL11 was analysed through immunofluorescence microscopy. As shown in Fig. 7, clear evidence was provided that there was interaction between CXCR3 and CXCL11 in conA-induced damage. In non-conA mice, CXCR3- and CXCL11-positive cells were separated from each other indicating minimal interaction (Fig. 7). Numbers of CXCL11- and CXCR3-positive cells were significantly increased compared with non-conA kidneys 8 h after conA treatment (Fig. 7). CXCL11-positive cells were mostly expressed in tubular epithelial cells, and the CXCR3-positive cells were bound to CXCL11-positive cells. These findings suggest that the production of CXCL11 by the tubular epithelium is responsible for the recruitment of CXCR3-positive cells. PF suppressed the interactions of CXCL11 and CXCR3.

## Discussion

In this study, it was demonstrated that PF protected mice from renal damage in conA-induced injury, and the mechanism of PF was shown to be closely associated with its suppressive effect on the CXCR3/CXCL11 chemokine axis in macrophage recruitment.

Intravenous injection of conA (at a dose of more than 10 mg/kg body weight) in mice can cause severe liver injury with high mortality rate [12], which is similar to acute hepatic failure. Experimental and clinical studies have demonstrated that liver diseases with liver dysfunction, especially in hepatic failure, are frequently accompanied by acute kidney injury (AKI) [13]. For example, renal damage consists of proximal tubular nephrosis in acute hepatic coma [14]. Furthermore, patients with acute hepatic failure usually suffer renal damage with higher levels of creatinine [14]. The kidney was damaged with higher tryptophan levels in the lipopolysaccharide (LPS)-induced acute hepatic failure model [15]. All these reports confirm that the kidneys are damaged in acute hepatitis. The data presented here also shows that BUN and Cr increases significantly after 8 h of conA administration. Renal tubular necrosis and inflammatory cell infiltration were also observed by HE staining. From these results, it could be concluded that the kidneys were also damaged in conA-treated mice.

ConA-induced liver injury in mice is characterised by inflammatory infiltration of macrophages, neutrophils, and T cells into the liver [16]. It has been shown that conA-induced liver injury depends on the production of inflammatory cytokines and chemokines, such as tumour necrosis factor-α (TNF-α) and IL1β [17]. Thus, macrophage infiltration has been considered to be a hallmark of all forms of injury [4, 18]. Macrophage deletion causes a decrease in pro-inflammatory cytokines and liver injury is suppressed in the conA mouse model [19]. These results demonstrate that macrophages are involved in the production of pro-inflammatory cytokines and played an important role in conA-induced liver injury [20]. It was reported that one of the initial events of renal disease was macrophage infiltration into the kidney [21, 22]. The results showed that CD68-positive macrophages increased significantly in kidneys 8 h after conA administration, meanwhile, the inflammatory cytokines such as IL1β and MCP1 increased notably in the damaged kidneys. However, surprisingly, the results showed that IL1β and MCP1 originated mostly from tubular epithelial cells, and not macrophages. These results are consistent with other reports [23, 24].

CXCR3 was not expressed in tubular epithelial cells, unlike IL1β and MCP1, and the immunofluorescent staining demonstrated that almost all of the macrophages could express CXCR3. This result indicates that CXCR3 may play an important role in macrophage infiltration. It has been suggested that CXCR3 and its ligands is one of the most important chemokine axes that promotes the arrival of cells into inflamed tissues [11]. This receptor interacts with three ligands: CXCL9, CXCL10, and CXCL11 [25]. However, in our preliminary experiments using real-time PCR analysis, CXCR9 and CXCR10 did not significantly increase 8 h after conA stimulation compared with non-conA mice (data not shown here), indicating that CXCL9 and CXCL10 may not be important in conA-induced renal damage. CXCL11 increased more than seven-fold in conA-vehicle mice compared with non-conA mice. Importantly, CXCL11 binds to CXCR3 with a much higher affinity than it does CXCL9 and CXCL10 [25]. Our results showed that CXCL11 was strong stained in tubular epithelial cells and was bound to CXCR3 after conA stimulation. Combined with these results it could be demonstrated that the CXCR3/CXCL11 signalling axis was over-activated and played a key role in macrophage infiltration in the kidneys in conA-treated mice.

Paeoniflorin is one of the principal bioactive components derived from the root of *P. lactiflora Palls/Paeoniae Radix*, a traditional Chinese herbal medicine which has been widely used in the treatment of liver and renal diseases. It has been reported that PF is widely used in the treatment of central nervous system diseases and serves as an antioxidant to protect neurons against oxidative stress [5]. PF is also able to alleviate acute lung injury, and the underlying mechanisms are probably attributed to a decrease in the production of pro-inflammatory cytokines through down-regulating the activation of p38, JNK, and NF-κB pathways in lung tissues [26]. We previously reported that PF administration attenuated DMN-induced liver fibrosis by regulating macrophage activation in the main organs [9]. In this study, PF had positive effects on liver function and renal function, with histopathological improvement at doses of 30 mg/kg and 150 mg/kg. Pre-treatment with PF significantly reduced macrophage infiltration and expression of pro-inflammatory cytokines IL1β and MCP1. PF inhibited the elevated expression of CXCR3 and CXCL11 and also suppressed the interactions between CXCR3 and CXCL11.

## Conclusion

In summary, the data presented in this study demonstrates that PF has a protective effect on conA-induced renal damage in mice, and this effect may be attributed, at least in part, to its inhibition of CXCR3/CXCL11-mediated macrophage infiltration.
